# Identification and characterization of PhbF: A DNA binding protein with regulatory role in the PHB metabolism of *Herbaspirillum seropedicae *SmR1

**DOI:** 10.1186/1471-2180-11-230

**Published:** 2011-10-14

**Authors:** Marco AS Kadowaki, Marcelo Müller-Santos, Fabiane GM Rego, Emanuel M Souza, Marshall G Yates, Rose A Monteiro, Fabio O Pedrosa, Leda S Chubatsu, Maria BR Steffens

**Affiliations:** 1 Department of Biochemistry and Molecular Biology, Universidade Federal do Paraná, Curitiba - PR, Brazil; 2 Department of Medical Pathology, Universidade Federal do Paraná, Curitiba - PR, Brazil

## Abstract

**Background:**

*Herbaspirillum seropedicae *SmR1 is a nitrogen fixing endophyte associated with important agricultural crops. It produces polyhydroxybutyrate (PHB) which is stored intracellularly as granules. However, PHB metabolism and regulatory control is not yet well studied in this organism.

**Results:**

In this work we describe the characterization of the PhbF protein from *H. seropedicae *SmR1 which was purified and characterized after expression in *E. coli*. The purified PhbF protein was able to bind to eleven putative promoters of genes involved in PHB metabolism in *H. seropedicae *SmR1. *In silico *analyses indicated a probable DNA-binding sequence which was shown to be protected in DNA footprinting assays using purified PhbF. Analyses using *lacZ *fusions showed that PhbF can act as a repressor protein controlling the expression of PHB metabolism-related genes.

**Conclusions:**

Our results indicate that *H. seropedicae *SmR1 PhbF regulates expression of *phb*-related genes by acting as a transcriptional repressor. The knowledge of the PHB metabolism of this plant-associated bacterium may contribute to the understanding of the plant-colonizing process and the organism's resistance and survival *in planta*.

## Background

Polyhydroxyalkanoates (PHA) are aliphatic polyesters biosynthesized by several bacteria [1-4] as a means of carbon storage and a source of reducing equivalents when other nutrients are limiting. The most frequently PHA produced is poly(3-hydroxybutyrate) or PHB [2]. The ability to produce PHB has been correlated with improved survival under stress conditions or in competitive environments [5,6]. PHB is generally produced in conditions of carbon oversupply and low levels of other nutrients such as nitrogen, phosphate or oxygen [7]. The biosynthesis of PHB is dependent on the activity of the following enzymes: (i) a 3-ketothiolase which condenses two acetyl-CoA yielding acetoacetyl-CoA (encoded by *phbA*), (ii) a NADPH-dependent acetoacetyl-CoA reductase which reduces acetoacetyl-CoA to (R)-3-hydroxybutyryl-CoA (encoded by *phbB*) and (iii) the PHB synthase (encoded by *phbC*) that catalyses the polymerization of (R)-3-hydroxybutyryl-CoA to form the polymer [8,9]. This polymer is stored intracellularly as insoluble inclusion bodies called PHB granules [1] which also contain about 2% protein as well as phospholipids [10]. The main protein associated with the PHB granules is phasin (encoded by *phaP*) which prevents coalescence of PHB granules by coating the granule surfaces [11-14]. However, other proteins have also been found associated with the granules, including transcriptional regulators such as PhaF from *Pseudomonas oleovorans *GPo1, PhaR from *Paracoccus denitrificans*, and PhaR from *Ralstonia eutropha *H16 [15-17].

Expression of enzymes involved in PHA/PHB biosynthesis and the granule-associated phasin are reported to be regulated at the transcriptional level [15,16,18-26]. This regulation may include repressors as well as activators [21]. The proteins PhbR from *Azotobacter vinelandii *UW136 [22] and PhaD from *Pseudomonas putida *KT2442 [24] are transcription activators. In contrast, PhaR of *P. denitrificans *represses *phaR *expression by binding to a TGC rich region which overlaps the -35/-10 promoter [16]. In *R. eutropha *H16 the PhaR protein binds to the -35/-10 *phaP *promoter at two sites: the transcriptional start site and upstream from the -35 at the promoter region, thereby blocking RNA polymerase [17]. The PhaR binding site determined in *R. eutropha *comprises two 12 bp repeated sequences not related to those observed in *P. denitrificans*, suggesting that DNA-binding sites for PhaR recognition and the mechanisms of regulation may vary.

The β-Proteobacterium *Herbaspirillum seropedicae *SmR1 is a plant-endophytic diazotroph found in association with economically important graminaceous species such as sugar cane, sorghum, rice and maize [27]. *H. seropedicae *SmR1 has been already described as a PHB producer using glucose as carbon source [28], however the molecular aspects of its PHB metabolism have not been addressed. The *H. seropedicae *SmR1 genome sequence analysis indicated several genes likely to be involved in PHB metabolism, including, *phbA, phbB *and *phbC *encoding for 3-ketothiolase, acetoacetyl-CoA reductase and poly(3-hydroxybutyrate) synthase. The *phbF *gene encoding a putative regulator was located downstream from *phbCB *[29]. In this work we characterized the transcriptional regulator PhbF of *Herbaspirillum seropedicae *SmR1.

## Methods

### Strains and plasmids

All bacterial strains and plasmids used in this work are listed in Table 1.

**Table 1:** Strains and plasmids used in this work

Strains	Relevant genotype	Reference/source
***E. coli ***		

BL21(DE3)	*hsdS gal (λcIts *857 *ind1 Sam*7 *nin*5 *lac*UV5-T7 gene 1).	Invitrogen
ET8000	*rbs lacZ::IS1 gyrA hutCc k *(wild-type).	[42]

***H. seropedicae ***		

SmR1	Wild-type, Nif^+^, Sm^R^.	[43]

**Plasmids**		

pET-28a	Expression vector, T7 promoter, Km^R^.	Novagen
pDK6	Expression vector *tac *promoter *lacIq*, Km^R^.	[44]
pKADO3	*H. seropedicae *SmR1 *phbF *cloned into pET-28a; expresses the His-tag PhbF protein.	This work
pKADO5	353 bp containing *phbF *promoter region cloned into pMP220 resulting in the *phbF*:: *lacZ *transcriptional fusion.	This work.
pMMS31	Derivative of pDK6 encoding PhbF from *H. seropedicae *SmR1.	This work.
pMMS35	381 bp containing *phaP1 *promoter region cloned into pMP220 resulting in the *phbP1*:: *lacZ *transcriptional fusion.	This work.
pMP220	Vector used to construct transcriptional *lacZ *fusions; Tc^R^.	[32]

### Media and growth conditions

*Escherichia coli *strains were grown in LB or M9 minimal media at 37°C [30]. The *H. seropedicae *SmR1 strain was grown at 30°C in NFbHPN-Malate medium supplemented with 20 mM NH_4_Cl [31]. Antibiotics were added as follows: ampicillin 100 μg.mL^-1^, tetracycline 10 μg.mL^-1^, streptomycin 20 μg.mL^-1 ^(*E. coli*) or 80 μg.mL^-1 ^(*H. seropedicae *SmR1), kanamycin 50 μg.mL^-1 ^(*E. coli*) or 500 μg.mL^-1 ^(*H. seropedicae *SmR1), chloramphenicol 30 μg.mL^-1 ^(*E. coli*) or 150 μg.mL^-1 ^(*H. seropedicae *SmR1) and nalidixic acid 10 μg.mL^-1^.

### Plasmid Construction

The *phbF *gene was amplified from the *H. seropedicae *SmR1 genome using the primers 5'GACTGGACTTCATATGACTACTGC3' and 5'CAACAGGATCCGGCAGAATG3' carrying NdeI or HindIII restriction sites (underlined). The amplified product was cloned into pET-28a to yield plasmid pKADO3, which over-expresses the PhbF protein fused to an N-terminal six-histidine tag (His-PhbF). To express PhbF from a *tac *promoter, *phbF *was obtained in an XbaI/HindIII fragment from pKADO3 and cloned into pDK6, yielding plasmid pMMS31.

### *Construction of transcriptional fusions *phbF::lacZ *and *phaP1::lacZ

The promoter regions of *phbF *(containing 353 bp including 54 bp of the *phbF *coding sequence) and *phaP1 *(containing 381 bp including 28 bp of the *phaP1 *coding sequence) were amplified from the *H. seropedicae *SmR1 genome and cloned into pMP220 [32], upstream from the promoter-less *lacZ *gene to yield the respective plasmids pKADO5 and pMMS35.

### β-galactosidase activity assay

β-galactosidase activity was determined in *E. coli *ET8000 carrying transcriptional fusion plasmids (pKADO5 or pMMS35), in the presence or absence of plasmid pMMS31 (expresses PhbF), grown in M9 minimal medium as described [33]. Protein concentration was determined using the Bradford method [34] with bovine serum albumin as standard.

### In silico *identification of DNA motif*

The MEME program [35] was used to detect a common motif among promoter regions of genes related to PHB metabolism in the *H. seropedicae *SmR1 genome [29]. The MEME program was set to identify not more than one motif with 6 to 50 bp in length. The conserved motif was represented in the LOGO format

### Purification of His-PhbF

*E. coli *strain BL21 (DE3) carrying pKADO3 was grown in LB medium at 37°C to an OD_600 _of 0.6-0.8. The culture was then induced with 0.5 mmol/L IPTG at 20°C for 15 hours. After harvesting, cells were lysed by sonication in buffer A (100 mmol/L NaCl, 50 mmol/L Tris-HCl pH 7.5, 10 mmol/L imidazole and 0.05% Triton X-100). After clarification by centrifugation at 14000 × *g *for 30 minutes at 4 °C, the protein extract was loaded onto a Hi-Trap Chelating Ni^2+ ^column (GE Healthcare). Protein elution was carried out using a linear imidazole gradient, and His-PhbF was eluted with 300 mmol/L imidazole in buffer A. Protein fractions were pooled and, after dialysis against buffer A with 50% glycerol, were stored in liquid N_2_.

### Electrophoretic Mobility Shift Assay (EMSA)

The promoter regions of genes related to PHB biosynthesis were amplified using fluorescent (VIC and FAM) end-labeled primers. Alternatively, *phbF *and *phaP1 *promoters were amplified and end-labeled using [^32^P]γ-ATP and T4 polynucleotide kinase [30]. DNA-binding assays were performed in 10 μL containing 20 nmol/L of end-labeled DNA, 100 ng of calf thymus DNA, and increasing amounts of purified His-PhbF in binding buffer (10 mmol/L Tris-HCl pH 7.5, 80 mmol/L NaCl, 1 mmol/L EDTA, 10 mmol/L β-mercaptoethanol and 5% (m/v) glycerol) following incubation at 30°C for 5 minutes. The fluorescent DNA was observed after excitation with UV light (254 nm) and the [^32^P]-labeled DNA was detected using a PhosphorImager screen and a STORM scanner.

### DNaseI footprinting assay

A 325bp DNA fragment containing the *phbF *promoter region was amplified using [^32^P]-labeled primer and genomic DNA as template [30]. The fragment was purified using the Wizard kit (Promega) and then incubated with His-PhbF in 50 mmol/L Tris-acetate pH 8.0, 8 mmol/L magnesium acetate and 10 mmol/L KCl at 30°C for 5 minutes. For partial hydrolysis, 1 unit of DNaseI (Invitrogen) was added and the reaction incubated at 30°C for 1 minute. The reaction was stopped by adding 0.2 volume of 0.5 mmol/L EDTA and heating at 80°C for 5 minutes. After ethanol precipitation of DNA fragments in the presence of yeast tRNA, samples were solubilized in 6 μL of loading buffer (47% formamide (v/v), 10 mmol/L EDTA, 0.05% bromophenol blue (m/v), 0.05% xylene xyanol (m/v)), denatured at 80°C for 5 minutes and loaded on a 6% (m/v) polyacrylamide denaturing DNA sequencing gel [30]. The *phbF *promoter region was sequenced using the T7 sequencing kit (GE Healthcare). Images of the autoradiograms were obtained using a PhosphorImager screen and a Storm scanner. Densitrometric profiles were analyzed using the ImageQuant v.5.2 program (Molecular Dynamics).

### Extraction of PHB granules

PHB granules were extracted from *H. seropedicae *SmR1 grown in NFbHP-malate medium containing 5 mM glutamate at 30°C until OD_600 _= 1.0, following a described procedure [36]. After extraction, granules were washed twice with water and then with acetone. Granules were dried under a nitrogen gas stream at room temperature and stored at -20°C.

### PHB granule-binding of the His-PhbF protein

The PHB granule-binding reaction was performed as described [37] with modifications. His-PhbF (25 μg) was incubated with 1 mg of purified *H. seropedicae *SmR1 PHB granules in a final volume of 100 μL in 50 mmol/L Tris-HCl pH 7.5. Samples were incubated at 37°C for 10 minutes and then centrifuged at 10,000 × g for 1 minute. The supernatant was collected and the granules were washed twice with 400 μL of 50 mM Tris-HCl pH 7.5 and the supernatant from each wash step was also collected separately. Protein bound to the granules was dissociated by incubation in 2% (m/v) SDS, 10% (m/v) glycerol and 5% (m/v) β-mercaptoethanol at 90°C for five minutes. Samples were analyzed by SDS-PAGE [38].

## Results and discussion

The *H. seropedicae *SmR1 PhbF protein was first identified in the cellular proteome by [39] using late log phase culture grown under ammoniotrophic conditions. The *phbF *gene (*H_sero2997*) is located downstream from *phbC *and *phbB *(GenBank: CP002039) and encodes a 188 amino acids protein with high similarity to *R. eutropha *H16 PhaR (183 amino acids, 83% identity, 90% similarity) [17], and, to a lesser extent, to *Rhodobacter sphaeroides *FJ1 (41% identity and 59% similarity) and *P. denitrificans *PhaR (restricted to the N-terminus with 37% identity and 56% similarity to the first 120 amino acids). *In silico *analysis indicated a helix-turn-helix motif located at its N-terminal sequence suggesting that PhbF is capable of DNA-binding and may act as a regulator of PHB biosynthesis genes in *H. seropedicae *SmR1.

To characterize the *H. seropedicae *SmR1 PhbF protein, it was overexpressed and purified as a His-tag fusion form (His-PhbF) from *E. coli *BL21(DE3) harboring the plasmid pKADO3 (Table 1). Most of the expressed His-PhbF was found in the soluble protein fraction when cells were induced at low temperature (20°C) and lysed in buffer containing Triton X-100 0.05% (m/v). This detergent at low concentration yielded a homogenous His-PhbF protein solution of 98% purity by Ni^2+^-affinity chromatography. Circular dichroism analysis indicated that purified His-PhbF is folded in the presence of the detergent (Additional file 1, Figure S1). Also, gel filtration chromatography indicated that *H. seropedicae *SmR1 PhbF is tetrameric in solution with an apparent molecular weight of 104.3 kDa (Additional file 1, Figure S2). The PhaR from *P. denitrificans *is also a tretrameric protein of approximately 95 kDa in solution [16].

**Figure 1:**
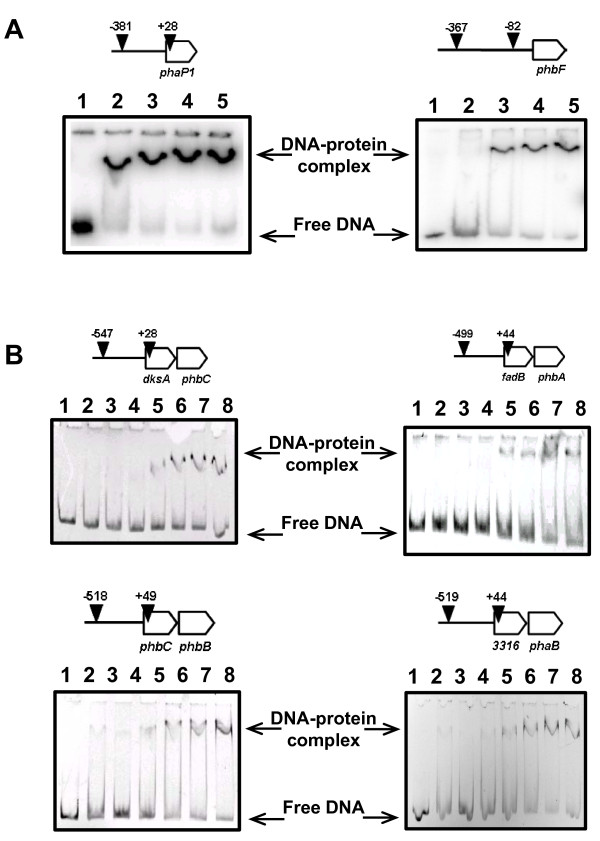
**The DNA-binding assays of purified His-PhbF from *H. seropedicae *SmR1 to the promoter regions of *phaP1, phbF, dskAphbC, fadBphbA, phbCphbB *and *H_sero3316phaB *were performed as described in Material and Methods**. DNA promoter regions used in the assays are indicated by vertical black arrow heads and numbers indicate base position related to the translation start of each gene. **Panel A: **DNA labeled with [^32^P]. Lanes 1 to 5 indicate increasing amounts of purified His-PhbF (0, 280, 570, 860 or 1100 nM). **Panel B: **Fluorescent labeled DNA. Lanes 1 to 8 indicate increasing amounts of purified His-PhbF (0, 62, 125, 250, 500, 750, 1000 or 1250 nM). Protein concentrations were calculated assuming His-PhbF as a tetrameric protein.

Twelve putative promoter regions were identified by DNA sequence analyses of genes potentially involved in PHA metabolism in *H. seropedicae *SmR1 (GenBank: CP002039, [29]) as shown in Additional file 1, Figure S3. All of these putative promoter regions, with the exception of *phaP2*, were assayed for DNA binding by His-PhbF. DNA band-shift assays showed that purified His-PhbF was able to bind specifically to these eleven promoter regions (Figure 1 and results not shown) but not to the unrelated *nifB *promoter [40](Additional file 1, Figure S4) indicating that the protein is active. The apparent dissociation constants observed varied from 150 nM (*phaP1*) to 450 nM (*phbF*).

**Figure 2:**
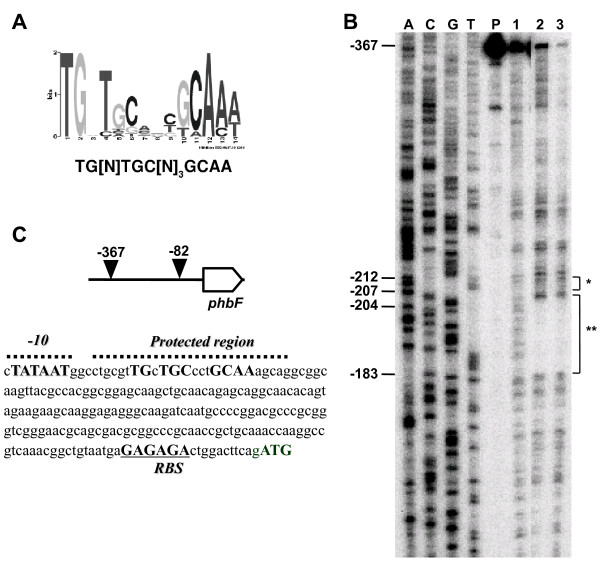
**Panel A: Sequence logo representing the consensus sequence of *pha *promoter regions identified by the program MEME motif discovery tool**. In the y axis the information is represented in bits indicating the nucleotide frequency in the sequence at that position. The putative consensus sequence probably recognized by PhbF is indicated. **Panel B: **DNase I-protection footprinting assay was carried out as described in Material and Methods. The non-coding strand of the *phbF *promoter was used as a probe. The assays were in the absence (lane 1) or presence 155 (lane 2) or 312 nM (lane 3) of the purified His-PhbF tetramer. Lane P indicates the undigested promoter region. The DNA sequencing reaction is indicated in lanes A, C, G, and T. The region showing protection from DNaseI digestion is indicated by **. The probable σ^70 ^promoter is indicated by *. Numbers indicate base position corresponding to the translation start codon.

These twelve promoter regions (including *phaP2*, additional file 1, Figure S3) were also analyzed *in silico *using the MEME program [35] which indicated the sequence TG[N]TGC[N]_3_GCAA as a probable DNA-binding motif for PhbF (Figure 2A). A similar sequence (CTGC[N]_3_GCAG) was also described in *R. sphaeroides *FJ1 as the DNA-binding site for the regulator PhaR [41]. Both sequences show two highly conserved triplets (TGC and GCA) which seem to be essential for DNA-binding of *R. sphaeroides *PhaR [41].

To verify if the TG[N]TGC[N]_3_GCAA sequence is important for DNA-binding of *H. seropedicae *SmR1 PhbF, a DNaseI footprinting assay was performed using the *phbF *promoter region and purified His-PhbF (Figure 2B). A clear DNaseI protection site was observed when His-PhbF was present in the assay. The protected site covers positions 181 to 204 upstream from the translation start site indicating that His-PhbF binds to a 24 bp region of its own promoter which includes the conserved TG[N]TGC[N]_3_GCAA motif indicated by the MEME program, reinforcing the suggestion that it is the DNA site recognized by the *H. seropedicae *SmR1 PhbF. Furthermore, a putative sigma 70-dependent promoter was also identified upstream from the PhbF DNA-binding site (position 208 to 212 from the translation start site) (Figure 2C). The proximity of both sites also suggests that *H. seropedicae *SmR1 PhbF may repress its own expression.

**Figure 3:**
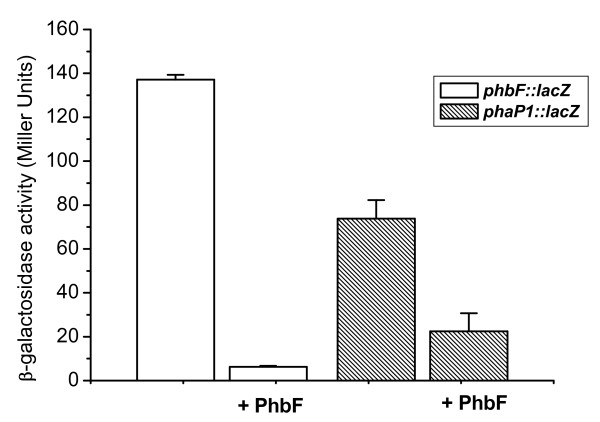
**β-galactosidase activity of *E. coli *strain ET8000 carrying *phbF::lacZ *or *phbP1::lacZ *fusion (plasmids pKADO5 and pMMS35, respectively)**. Assays were performed as described in Material and Methods. The His-PhbF protein was expressed by the *tac *promoter from the plasmid pMMS31. Data represents the average ± standard deviation of at least three independent determinations. Background activity of cells carrying pMP220 (control vector) in the presence of pMMS31 was less than 6 Miller units.

We verified the potential repressor activity of PhbF in *E. coli *ET8000 by using a gene reporter expression assay with *phaP1 *and *phbF *promoters fused to the *lacZ *gene. These genes were chosen because they have the putative PhbF-binding sequence highly similar to the consensus sequence, and also because EMSA assay showed clear interaction with these promoters. The β-galactosidase activities indicated that both *phaP1 *and *phbF *promoters were functional in *E. coli *(Figure 3). However, a clear decrease in β-galactosidase activity is observed if *H. seropedicae *SmR1 PhbF is present (expressed upon plasmid pMMS31), indicating that PhbF represses the expression of the phasin gene (*phaP1*) and also of its own gene promoter (*phbF*). Expression of an unrelated protein (NifH) did not affect β-galactosidase activity of *E. coli *bearing the *phbF::lacZ *and *phaP1::lacZ *fusions (data not shown), reinforcing the repressor effect of PhbF.

**Figure 4:**
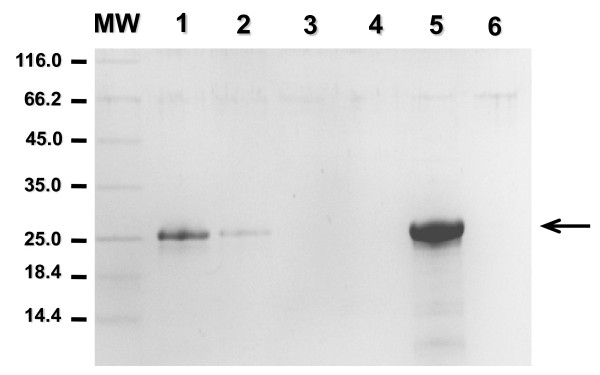
**Binding of His-PhbF to PHB granules**. Purified His-PhbF was incubated with polyhydroxybutyrate (PHB) granules as described in Material and Methods. Lane 1: 2 μg of purified His-PhbF; lane 2: non-adsorbed protein; lanes 3 and 4: washing buffer; lane 5: PHB-adsorbed protein after elution with 2% (m/v) SDS, 10% (m/v) glycerol and 5% (m/v) β-mercaptoethanol at 90°C for five minutes; lane 6: PHB-granule control treated with 2% (m/v) SDS, 10% (m/v) glycerol and 5% (m/v) β-mercaptoethanol at 90°C for five minutes. MW: molecular weight markers (kDa). Arrow indicates His-PhbF. The SDS-PAGE gel was stained with Coomassie blue.

Protein domain analysis indicated that PhbF contains a DNA-binding motif and a domain possibly involved in binding PHB. Therefore, we tested if *H. seropedicae *SmR1 PhbF was able to interact with PHB granules *in vitro*. The purified His-PhbF was incubated with PHB granules extracted from *H. seropedicae *SmR1 and the protein remaining in solution was visualized by SDS-PAGE (Figure 4). When His-PhbF was incubated with PHB granules most of the protein was extracted from solution (Figure 4, lane 2). The protein remained bound to the granule even after two washing steps (lanes 3 and 4), and was released only after heating in the presence of SDS, indicating a strong interaction between His-PhbF and PHB.

Our results indicate that *H. seropedicae *SmR1 PhbF is capable of DNA binding and also of associating with PHB granules. In addition, expression of PhbF from *H. seropedicae *SmR1 leads to 10 and 4-fold reduction (P < 0.05) in expression of *phbF *and *phaP1 *promoters, respectively. These results strongly suggest that *H. seropedicae *SmR1 PhbF protein is a repressor which controls expression of genes involved in PHB production as well its own expression. In both respects it shows similarity with the PhaR regulator from *R. eutropha *[17] and from *P. denitrificans *[16].

The expression of *phbF *gene in *H. seropedicae *SmR1 increases sharply in the log phase (not shown) and PHB starts to accumulate in the log phase reaching maximum as the culture entry in the stationary phase [28], suggesting that the repressor activity of PhbF may be relieved as PHB oligomers levels increase inside the cell, as suggested in *R. eutropha *and *P. denitrificans *[11,16,17]. The expression of *phaP1 *has a similar pattern. We hypothesize that when PHB oligomers levels increase, the PhbF protein is sequestred, allowing transcriptional initiation. Whether PhbF can be released from DNA by binding to PHB, thus allowing expression of *pha/phb *genes once PHB synthesis is favored is not known.

The production of reserve material such as PHB has important metabolic features, since stress endurance and survival is improved when bacteria produce PHB, as observed for *Azospirillum brasilense *[5], and cells with high PHB content were able to increase the population 2-3 fold and survive for longer periods of starvation as seen in *Sinorhizobium meliloti *[6]. Therefore, knowledge of the PHB metabolism of plant-associated bacteria may contribute to the understanding of the colonization process and improvement of their resistance and survival under colonizing conditions.

## Conclusions

Our results show that PhbF from *H. seropedicae *SmR1 binds to eleven promoter regions of genes related to PHB metabolism. A DNA-binding consensus sequence was determined and confirmed by DNase-I footprinting assay. Furthermore, expression of *phbF::lacZ *and *phaP1::lacZ *fusions indicated that PhbF may act as a transcriptional repressor of genes involved in PHB metabolism in *H. seropedicae *SmR1.

## Authors' contributions

MASK carried out cloning, expression, purification and EMSA of PhbF, participated in experimental design and drafted the manuscript. MMS carried out cloning, *in vivo *assays, participated in experimental design and drafted the manuscript. FGM carried out the DNase I-protection footprinting assay. RAM participated in DNA sequence analysis. EMS, FOP and LSC participated in experimental design, discussion and manuscript writing. MGY participated in manuscript drafting and correction. MBRS conceived of the study and participated in its design and coordination. All authors read and approved the final manuscript.

## Supplementary Material

Additional file 1 **Figure S1: Circular dichroism spectrum of purified *H. seropedicae *His-PhbF**. Figure S2: Gel filtration chromatography of purified *H. seropedicae *His-PhbF. Figure S3: Schematic organization of genes probably involved in polyhydroxyalkanoate (PHA) pathway and regulation in *H. seropedicae*. Figure S4: The DNA-binding assays of purified His-PhbF from *H. seropedicae *to the *nifB *promoter region (negative control). Click here for file
