# Supplementary material for: Identification and characterization of PhbF: A DNA binding protein with regulatory role in the PHB metabolism of Herbaspirillum seropedicae SmR1
Source: BMC Microbiol. 2011 Oct 14;11:230. doi: 10.1186/1471-2180-11-230 (PMC3206438; doi:10.1186/1471-2180-11-230)
Supplement: Additional file 1 — Figure S1: Circular dichroism spectrum of purified H. seropedicae His-PhbF. Figure S2: Gel filtration chromatography of purified H. seropedicae His-PhbF. Figure S3: Schematic organization of genes probably involved in polyhydroxyalkanoate (PHA) pathway and regulation in H. seropedicae. Figure S4: The DNA-binding assays of purified His-PhbF from H. seropedicae to the nifB promoter region (negative control). [file 1471-2180-11-230-S1.DOC]

**Additional Material**

**
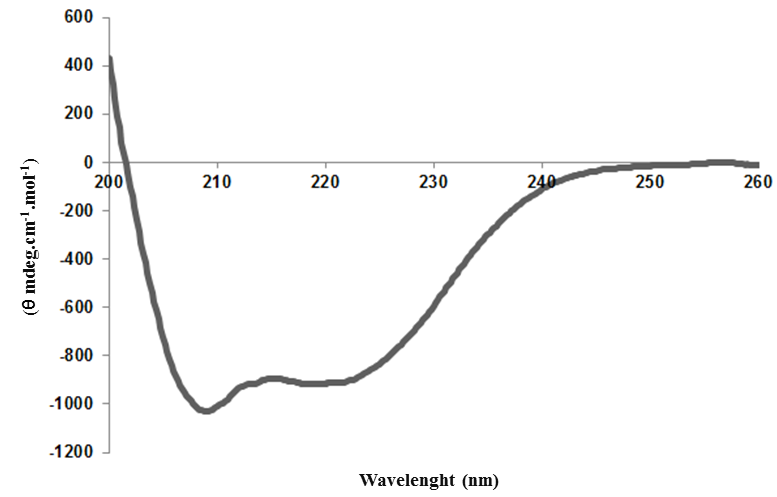
**

**Figure S1. Circular dichroism spectrum of purified *H. seropedicae* His-PhbF.** The UV spectrum (200-260 nm) was obtained with a protein concentration of 0.2 mg/mL in 20 mM phosphate buffer pH 7.5, 100 mM NaCl, 0.05% (m/v) of Triton X-100, at 25 ºC.

**
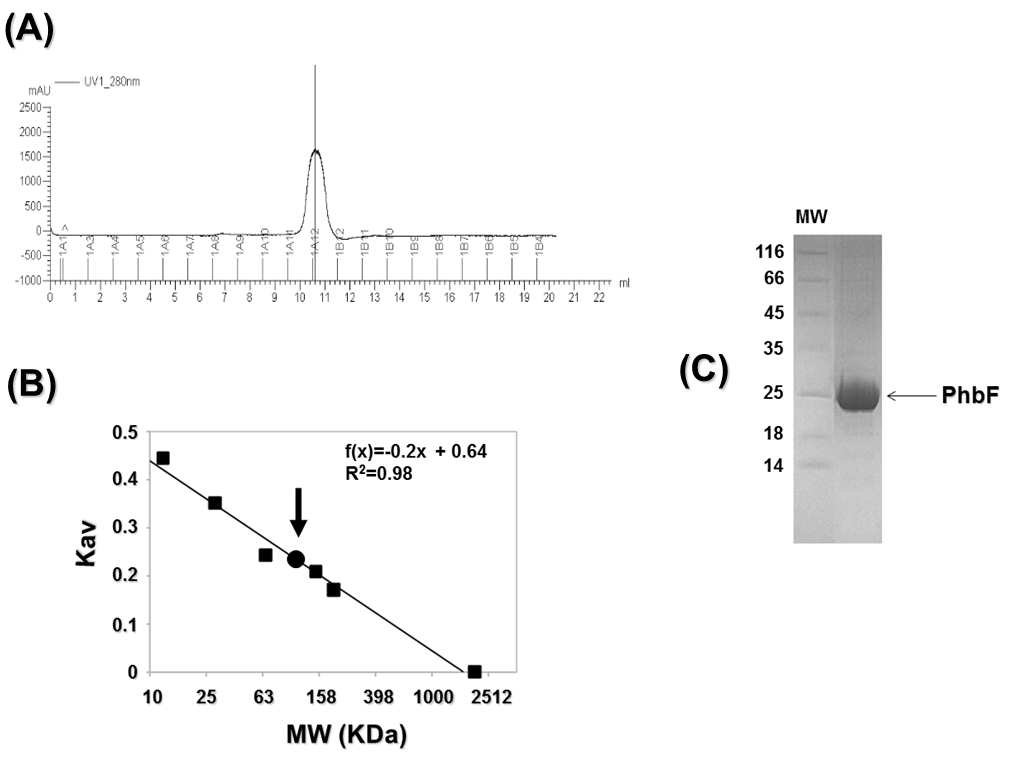
**

**Figure S2. Gel filtration chromatography of purified His-PhbF from *H. seropedicae*.** His-PhbF was overexpressed and purified as described in Material and Methods and 2.5 mg of purified protein was loaded onto a Superose 12 HR 10/30 column (GE Healthcare). (A) Protein elution profile was followed by UV absorption (280 nm). (B) Determination of molecular weight carried out using gel-filtration molecular weight markers (black dots): cytochrome C (12.4 kDa), carbonic anhydrase (29 kDa), bovine serum albumin (BSA) (66 kDa), alcohol dehydrogenase (150 kDa) and α-amylase (200 kDa) (GE Healthcare). Arrow indicates the His-PhbF. (C) Protein purity was also analyzed by SDS-PAGE stained with Coomassie-blue. MW indicates molecular weight markers (kDa).


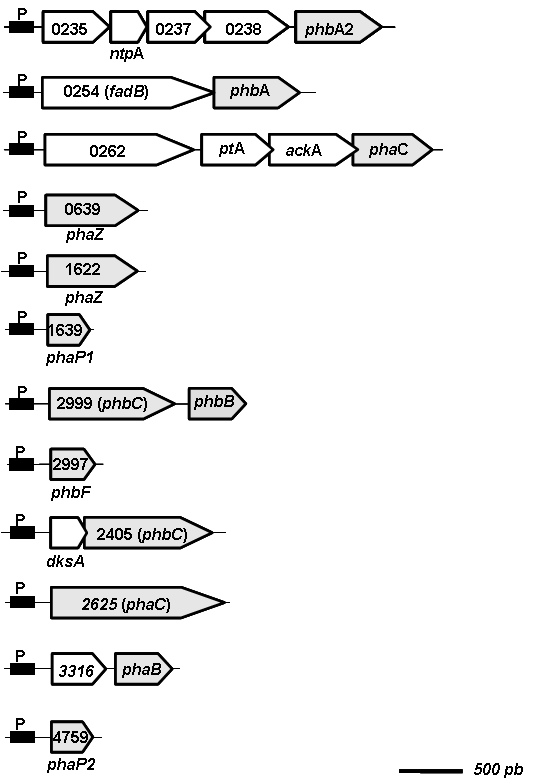


**Figure S3. Schematic organization of genes probably involved in polyhydroxyalkanoate (PHA) pathway and regulation in *H. seropedicae*.** Scheme shows the twelve promoter regions (black boxes) analyzed for DNA binding activity of purified His-PhbF. Arrows indicate genes and transcription orientation. *orfs* are indicated by numbers following the annotated *H. seropedicae* genome (GenBank accession number: CP002039). Gray arrows indicate annotated *pha/phb* genes: *phbA*: acetyl-CoA acetyltransferase; *phbB*: acetoacetyl-CoA reductase; *phbC*: poly-beta-hydroxyalkanoate synthase; *phaP:* phasin protein; *phaZ*: poly-beta-hydroxyalkanoate depolymerase; *phaB*: 3-ketoacyl-CoA reductase; *phaC*: poly(3-hydroxyalkanoate) synthetase; *phbF*: polybetahydroxybutyrate accumulation regulatory protein. Annotated genes located near *pha/phb* are indicated: *ackA*: acetate kinase protein; *dksA*: DnaK suppressor; *fadB*: 3-hydroxyacyl-CoA dehydrogenase oxidoreductase; *ntpA*: dATP pyrophosphohydrolase; *pta*: phosphate acetyltransferase.


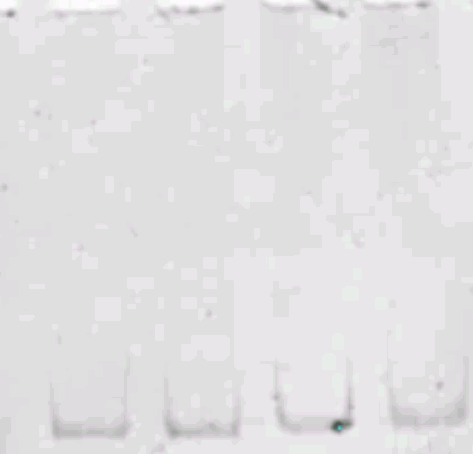


**1 2 3 4**


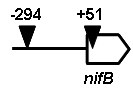


**Free DNA**

**Figure S4: The DNA-binding assays of purified His-PhbF from *H. seropedicae* to the *nifB* promoter region (negative control)***.* The *nifB* promoter region used is indicated by vertical black arrow heads with number indicating base position related to the translation start of the *nifB* gene. Fluorescent labeled DNA was assayed as described in Material and Methods. Lanes 1 to 4 indicate increasing amounts of purified His-PhbF (0, 62, 250 or 1250 nM, respectively). Protein concentrations were calculated assuming His-PhbF as a tetrameric protein.
